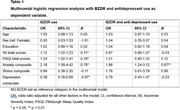# Benzodiazepine Use in Middle‐Aged Adults at Risk for AD

**DOI:** 10.1002/alz70860_096806

**Published:** 2025-12-23

**Authors:** Ivana Chan, Paul Maruff, Yen Lim

**Affiliations:** ^1^ Turner Institute for Brain and Mental Health, Melbourne, VIC, Australia; ^2^ Cogstate Ltd., Melbourne, VIC, Australia; ^3^ Turner Institute for Brain and Mental Health, School of Psychological Sciences, Monash University, Melbourne, VIC, Australia

## Abstract

**Background:**

Use of benzodiazepines has been identified as a potential risk factor of dementia, but research examining this association in midlife has been limited.

**Method:**

The current cross‐sectional study examined the associations between benzodiazepine use and demographic and clinical characteristics associated with their use in middle‐aged cognitively unimpaired adults (*n* = 4,088) with increased risk for dementia enrolled in the Healthy Brain Project. The study also investigated the associations between benzodiazepine use and dementia risk, as measured by CAIDE risk scores.

**Result:**

Benzodiazepine use was associated significantly with higher (worse) Insomnia Severity Index total scores, higher (worse) Pittsburgh Sleep Quality Index total scores, higher anxiety composite scores, and lower depression composite scores. For dementia risk, while the expected characteristics of age, sex, education level, severity of depressive symptoms and sleep difficulties all contributed independently to dementia risk in this sample of middle‐aged adults, BZDR use did not.

**Conclusion:**

These investigations showed that despite the increased risk for dementia in the HBP sample, current BZDR use, with or without concomitant antidepressant use, was not associated with quantitatively defined risk for dementia. Limitations include: 1) cross‐sectional nature of the study limited ability to ascertain causality; 2) individuals with major psychiatric conditions were excluded from the study, thus limiting external validity of the current findings; 3) HBP sample has a higher proportion of individuals with a family history of dementia compared to the general population, hence the sample presents with an increased risk of developing dementia and generalizability of our findings may be limited.